# Grazing by wild red deer can mitigate nutrient enrichment in protected semi-natural open habitats

**DOI:** 10.1007/s00442-022-05182-z

**Published:** 2022-05-12

**Authors:** Friederike Riesch, Anya Wichelhaus, Bettina Tonn, Marcus Meißner, Gert Rosenthal, Johannes Isselstein

**Affiliations:** 1 Grassland Science, Department of Crop Sciences, University of Goettingen, Göttingen, Germany; 2 Centre of Biodiversity and Sustainable Land Use, University of Goettingen, Göttingen, Germany; 3 Department of Landscape and Vegetation Ecology, University of Kassel, Kassel, Germany; 4 Department of Livestock Sciences, Research Institute of Organic Agriculture (FiBL), Frick, Switzerland; 5 Institut für Wildbiologie Göttingen und Dresden e.V., Göttingen, Germany

**Keywords:** Atmospheric deposition, *Cervus elaphus*, Natura 2000, Nitrogen, Phosphorus

## Abstract

Eutrophication through atmospheric nutrient deposition is threatening the biodiversity of semi-natural habitats characterized by low nutrient availability. Accordingly, local management measures aiming at open habitat conservation need to maintain habitat-specific nutrient conditions despite atmospheric inputs. Grazing by wild herbivores, such as red deer (*Cervus elaphus*), has been proposed as an alternative to mechanical or livestock-based measures for preserving open habitats. The role of red deer for nutrient dynamics in protected open habitat types, however, is yet unclear. Therefore, we collected data on vegetation productivity, forage removal, quantity of red deer dung and nutrient concentrations in vegetation and dung from permanent plots in heathlands and grasslands (eight plots à 225 m^2^ per habitat type) on a military training area inhabited by a large population of free-ranging red deer over one year. The annual nutrient export of nitrogen (N) and phosphorus (P) by red deer grazing was higher than the nutrient import through red deer excreta, resulting in an average net nutrient removal of 14 and 30 kg N ha^−1^ a^−1^ and 1.1 and 3.3 kg P ha^−1^ a^−1^ in heathlands and grasslands, respectively. Even when considering approximate local atmospheric deposition values, net nutrient depletion due to red deer grazing seemed very likely, notably in grasslands. Demonstrating that grazing by wild red deer can mitigate the effects of atmospheric nutrient deposition in semi-natural open habitats similarly to extensive livestock grazing, our results support the idea that red deer are suitable grazing animals for open habitat conservation.

**Supplementary Information:**

The online version contains supplementary material available at 10.1007/s00442-022-05182-z.

## Introduction

Although the EU member states committed themselves to prevent the deterioration of natural habitats already in 1992 (Council Directive 92/43/EEC, Article 6(2)), the state of nature in Europe continuously declines and the EU has failed to reach the objectives of its Biodiversity Strategy to 2020 (EEA 2020). Open habitat types, e.g., grasslands, which often depend on particular agricultural practices (Halada et al. 2011), show the most pronounced deteriorating trends and their area is decreasing both outside and inside protected areas of the European Natura 2000 network (EEA 2020).

While the intensification of agricultural practices as well as abandonment and succession into forest have been noted as the most prevailing pressures for open habitat types, the latest report by the European Environment Agency indicated that the impact of atmospheric emissions and air pollution have been underestimated so far (EEA 2020). Actually, large areas of Europe are subject to substantial deposition of nutrients released from anthropogenic activities (Hettelingh et al. 2017). The eutrophication through atmospheric nutrient deposition is severely threatening the biodiversity of semi-natural habitats, which are usually characterized by low nutrient availability. As a consequence, critical loads for nutrient deposition have been defined as thresholds below which plant communities should be safe from undergoing deposition-related changes (Bobbink et al. 2015). The effectiveness of any local conservation measure is hence to some extent conditioned by the ability to maintain habitat-specific nutrient conditions despite ineluctable atmospheric nutrient inputs.

Human activities have apparently caused a threefold increase in soluble nitrogen (N) deposition over global terrestrial areas from 1850 to the present (Kanakidou et al. 2016). The consequences of atmospheric N deposition, such as soil acidification (Horswill et al. 2008), increased plant productivity (Stevens et al. 2015), changes in plant species composition (Field et al. 2014; Dobben and Vries 2017; Peppler-Lisbach et al. 2020), decreased plant diversity (Bobbink et al. 2010) and ecosystem stability (Zhang et al. 2016) and cascading effects on ecosystem services (Clark et al. 2017) have received much attention. However, the atmospheric deposition of phosphorus (P) can also have severe ecological consequences (Mahowald et al. 2008; Camarero and Catalan 2012) and plant-available P can even be a more important driver for plant community dynamics and diversity patterns than N (Wassen et al. 2005; Ceulemans et al. 2014). Although the anthropogenic contribution to atmospheric nutrient budgets is smaller for P than for N, recent research highlights that human activities, being the source of ca. 30% of atmospheric P, affect the global P cycle much more than previously thought (Yuan et al. 2018; Pan et al. 2021). Moreover, P deposition in Europe has notably increased since the beginning of the twenty-first century and is therefore considered as a potentially important driver of plant productivity in ecosystems (Pan et al. 2021). Especially in landscapes where (semi-)natural ecosystems are interspersed in areas of intensive human land use, pervasive effects of P deposition through short-distance transfer can be expected (Tipping et al. 2014).

Increases in available nutrients reduce plant diversity as competition for light increases, but the biomass removal by herbivores can reduce light limitation and thereby foster biodiversity (Borer et al. 2014). Additionally, large herbivores themselves can be important agents in the nutrient cycling of N (Augustine et al. 2013; Schrama et al. 2013) as well as P (Schütz et al. 2006; Jewell et al. 2007). Foraging herbivores take up nutrients and transfer them in the form of dung and urine to potentially different locations, whereby P is almost exclusively excreted in dung and N in both dung and urine (Haynes and Williams 1993). N is regarded as a currency in herbivore forage selection (Langvatn and Hanley 1993; Berteaux et al. 1998), but there are also indications that herbivores select forage based on its P concentration (Dykes et al. 2018). While much research has focused on the role of N, measuring both N and P in the forage and excreta of herbivores is therefore advisable to understand the effects of herbivore-driven nutrient fluxes on plant communities (Sitters et al. 2017).

Nutrient transport by livestock on pasture has been studied intensively (e.g., Haynes and Williams 1993; Schnyder et al. 2010; Zhou et al. 2017a) and we know much about patterns in vegetation diversity and structure created by different livestock species at paddock-scale that play a crucial role for grassland biodiversity (e.g., Adler et al. 2001; Rook et al. 2004; Tonn et al. 2019). Fewer studies have addressed nutrient transport in larger systems composed of different vegetation types (Uytvanck et al. 2010; Koch et al. 2018; Pelve et al. 2020), or by wild herbivores (Schütz et al. 2006; Abbas et al. 2012). Native wild ungulates, in particular the widespread red deer (*Cervus elaphus*), have recently come into focus in Europe because grazing by free-ranging herbivores might be an alternative option for the conservation of open habitat types, especially in target areas not easily accessible to humans and livestock, such as military training areas or post-mining landscapes (Tschöpe et al. 2011; Müller et al. 2017; Riesch et al. 2019). The effects of grazing by wild red deer on the vegetation have been investigated in different settings revealing benefits for the understory species richness in boreal old-growth forests (Hegland et al. 2013; Hegland and Rydgren 2016), grassland diversity (Schütz et al. 2003; Riesch et al. 2020; Wichelhaus 2020) as well as the vitality of heathlands (Riesch et al. 2019). From an applied conservation point of view, biomass removal by red deer hence appears to be an effective measure for preserving open habitats and counteracting forest succession. We are, however, not aware that any attempt has been made to quantify the nutrient import and export by wild red deer for protected plant communities, such as the Natura 2000 habitat types defined by the European Habitats Directive (Council Directive 92/43/EEC). A quantitative assessment of nutrient fluxes would not only increase our understanding of the ecological function of the key-stone species red deer and its interactions with soil, vegetation and atmosphere, but would also enable us to weigh up the magnitude of nutrient fluxes by wild red deer relative to anthropogenic nutrient deposition in semi-natural habitat types.

To fill this knowledge gap, the present study combined data on vegetation productivity, forage quality, quantity of red deer dung and faecal nutrient concentrations, which were collected over one year from permanent plots in semi-natural open habitats on an active military training area in Central Europe, where a large population of wild, free-ranging red deer (estimated at ca. 7,000 animals in summer by the Federal Forestry Office) uses open habitats for foraging (Meißner et al. 2013; Reinecke et al. 2013). We focused on two habitat types protected under the European Habitats Directive: European dry heaths, a habitat type with generally low productivity and forage quality, and lowland hay meadows, with relatively high productivity and forage quality, for which a previous study had attested considerable rates of biomass removal by red deer (Riesch et al. 2019). We quantified the import and export of N and P by red deer in these open habitat types to address the following hypotheses:

- H1a) Wild red deer counteract atmospheric nutrient deposition in semi-natural open habitats because nutrient removal by grazing is high and import through excreta is low.
- H1b) Wild red deer augment atmospheric nutrient deposition in semi-natural open habitats because nutrient import through excreta is higher than nutrient removal through grazing.
- H2a) Higher forage removal by red deer in a habitat type with higher productivity and forage quality results in higher nutrient export and a larger difference between nutrient export and nutrient import by red deer than in a habitat type with a less attractive forage resource.
- H2b) The difference between nutrient export and nutrient import by red deer does not differ between habitat types of different productivity and forage quality because increased nutrient removal in the high-quality habitat is offset by increased excreta deposition.

Our study hence allows assessing the potential of wild red deer as agents for the conservation of semi-natural open habitats against the background of anthropogenic nutrient deposition and represents another step towards a holistic understanding of the role of wild red deer in ecosystem functioning.

## Methods

This article does not contain any studies with human participants or animals performed by any of the authors.

### Study area

We performed our study in the Grafenwöhr military training area (GTA) in Bavaria, Germany. Long-term (1981–2010) annual averages (± SE) of temperature and precipitation are 8.3 ± 0.04 °C and 701 ± 4 mm (calculated from data of four weather stations of the German Weather Service in the immediate vicinity of GTA). The area has been dedicated to military training since the beginning of the twentieth century and has been attributed the status as a Site of Community Importance within the Natura 2000 framework in 2004 (Site Code: DE6336301). Although the area (ca. 230 km^2^) is not fenced, public entrance is not allowed. GTA is mainly covered by forest (40%) and open habitats (30%), which are to some extent managed by mowing or mulching (Raab et al. 2019). Other parts of the open areas remain without mechanical management but might occasionally burn or be otherwise disturbed by military activities. The Federal Forestry Administration (Bundesforst) of the German Institute for Federal Real Estate is responsible for the land and wildlife management. Wildlife is abundant and especially the population density of red deer is high (hunting bag records for the period April 2015 to April 2016: 1678 red deer, 538 roe deer (*Capreolus capreolus*), 522 wild boar (*Sus scrofa*)). In the centre of GTA, the density of red deer is estimated at 25 individuals per km^2^. For decades, the Federal Forestry Administration applies an adapted wildlife management regime to reduce damage by red deer in forests. To this end, in the peripheral forests of GTA, hunting is practised during the entire hunting season from the beginning of June to the end of January, while hunting in the open habitats in the centre of GTA has been limited to few driven hunts in early winter. As a consequence, the red deer regularly access the open habitats for foraging even at daytime (Meißner et al. 2013; Richter et al. 2020). Nevertheless, the intensity of human activities (mostly military operations and traffic) influences the spatio-temporal use of open and covered habitats by red deer in GTA to some extent (Richter et al. 2020).

The focus of this study is on a grassland and a heathland habitat type (EU Habitats Directive Annex I habitat types 6510 lowland hay meadows, hereafter ‘grasslands’, and 4030 European dry heaths, hereafter ‘heathlands’). The grassland habitat type, covering a total area of ca. 330 ha, is situated in the western part of the GTA as part of a heterogeneous open landscape and is characterized by a diverse community of grass, forb and legume species (46 plant species per 25 m^2^ on average; Riesch et al. (2018)). The heathland habitat type is dominated by the dwarf shrub *Calluna vulgaris* and occurs mainly in the eastern part of GTA with a total area of ca. 450 ha (Online Resource Fig. S1). Both habitat types are characterized by low soil fertility, as military land use during the past century has prevented agricultural intensification (Riesch et al. 2018).

### Data collection

In this study, we combined different data sets that were compiled on six sampling dates in 2015 to 2016 (April, May, June, August, October 2015, and April 2016). Data on vegetation productivity and quality as well as forage removal by red deer were collected as part of the studies by Riesch et al. (2019, 2020). Dung data were collected by Wichelhaus (2020) investigating endogenous seed dispersal by red deer.

In each habitat type, we selected four sampling sites. As the studies of Riesch et al. (2019, 2020) included a comparison between different open habitat management regimes, grassland sampling sites were split into three different treatment areas (burnt, mown, untreated). The present study considers only the burnt (B) and untreated (U) treatment areas because no dung data were collected in the mown treatment, as mowing was expected to impact on the number of detectable pellet groups. Heathland sampling sites were split into two different treatment areas (B, U) corresponding to potentially applicable conservation management practices. As burning (performed in heathlands after the sampling date in August 2015) succeeded only on two sites, there were, effectively, two heathland sites in which the B treatment was burnt and two sites where the B treatment was not burnt. The different vegetation management treatments are, however, not in the focus of the present study and served only to formulate the random effects structure in the linear mixed-effects models (see below).

### Vegetation data

We collected the vegetation data on one plot (15 × 15 m) per sampling site and treatment. To quantify the export of nutrients through grazing, we first assessed the productivity of the grazed vegetation based on rising-plate meter measurements of the compressed sward height and calibration cuts (Correll et al. 2003) and movable exclusion cages (McNaughton et al. 1996). We then calculated the forage removal by red deer as the difference in biomass increments between the vegetation under the exclusion cage temporarily protected from grazing and the continuously grazed vegetation on the surrounding plot area. Second, we collected hand-pluck samples (imitating red deer foraging behaviour by cutting the upper third of the vegetation at different random spots per plot) on each sampling date to determine the concentrations of N and P in the plant material. For detailed information on the forage quantity and quality assessments see Riesch et al. (2019).

### Red deer dung data

To assess the quantity of red deer dung accumulated per sampling date, we applied the faecal accumulation rate method (Mayle et al. 1999; Marques et al. 2001; Alves et al. 2013). At the first sampling date in April 2015, we cleared plots of the same size as the vegetation plots (15 × 15 m) from all faeces. These dung plots were positioned adjoining the vegetation plots: per sampling site two dung plots were situated on opposing sides of the U treatment plot and one plot was placed next to the B treatment plot. At the following sampling dates, all red deer dung pellet groups were counted (including any dung groups lying at the plot edge) and subsequently removed from the plot to assess the respective number of pellet groups accumulated per plot between sampling dates.

We applied a correction factor to the number of accumulated dung pellet groups to correct for possible decay and disappearance of dung between sampling dates (Laing et al. 2003; Torres et al. 2013). As decomposition of dung is highly dependent on habitat and microclimate conditions (Mayle et al. 1999), we determined site-specific correction factors for each sampling period in a decay experiment. At each sampling site and date, five pellet groups as fresh as possible (shiny surface, no visible activity of coprophages) were placed at a representative location spaced at a distance of at least 2 m to each other and marked with a stick to ensure retrieval. The persistence of each pellet group was checked at the subsequent sampling date. It was assumed that a pellet group had decayed if less than six pellets were retrieved (Mayle et al. 1999). The number of pellet groups per sampling plot and date corrected by the decay rate (*pg*_cor_) was calculated as follows (Wichelhaus 2020):

$$pg_{{{\text{cor}}}} = \frac{{pg_{{{\text{obs}}}} }}{{1 - \frac{r}{2}}}$$

where *pg*_obs_ is the observed number of dung pellet groups and *r* is the fraction of pellet groups that had disappeared since the previous sampling date in the decay experiment. Hence, if no pellet groups had decayed between successive sampling dates *pg*_cor_ equaled *pg*_obs_.

As in the original dataset (Wichelhaus 2020), values for *r* and dung dry mass were only recorded in 2015, we acquired the missing values for the final study period (October 2015 to April 2016) from November 2020 to April 2021. These supplemental data were collected in the same sampling sites and according to the same procedure as the original data. To adequately account for possible decay during the long winter period, we checked the marked pellet groups in November in January, March and finally April. When checking in March, one pellet group in a grassland site had vanished and was replaced by a freshly collected pellet group. At the end of the winter decay experiment, one pellet group had vanished from a heathland site, while two pellet groups in total had decayed in grasslands.

To determine dung dry mass, at each sampling date five fresh pellet groups per sampling site were collected, dried at 65 °C for at least 24 h and then weighed. Dung dry mass data for the final study period were collected in April 2021. Additionally, we aimed to collect fresh red deer dung from five further pellet groups per sampling site and date for assessing dung nutrient concentrations. In this we did not always succeed because of low availability of fresh dung pellet groups.

### Laboratory analyses

After collection from the field, we dried all plant and dung samples at 60 °C for at least 24 h and milled them to 1-mm grain size. We determined the total N concentration in plant and dung samples applying Dumas combustion in a CN elemental analyzer (vario MAX cube, elementar, Langenselbold, DE, for data from 2015 and vario EL III, elementar, Langenselbold, DE, for data from April 2016). To evaluate if the diet consumed by red deer had a similar quality as the vegetation on our experimental plots, we additionally predicted the expected faecal N percentage based on the plant N concentration (Online Resource Appendix A1).

We analysed the concentrations of P in all samples after digestion with 65% HNO_3_ at 195 °C for 8 h by Inductively Coupled Plasma Optical Emission Spectrometry (6300 DUO ICP-OES, Thermo Fisher Corporation, Waltham, MA, US for data from June 2015 and Optima 3000 XL, Perkin Elmer, Waltham, MA, US, for all other data). All nutrient concentrations are given in percent dry mass.

### Data processing and statistical analyses

We conducted all data processing and statistical analyses in R version 4.0.3 (R Core Team 2020). To combine the plant and dung data, we calculated the arithmetic mean of the dung quantity data values from the two dung plots adjoining the U treatment plot per sampling site. Hence, our data set consisted of 80 observations for each response variable (4 sampling sites with 2 plots each in 2 habitat types, 5 periods).

For different reasons, our dataset contains a certain number of missing values. As burning in heathlands was successful only on two sampling sites, replication did not suffice for a meaningful biomass calibration, so that determining the vegetation productivity was not possible (cf. Riesch et al. 2019). For the two actually burnt heathland plots, data on nutrient export are hence not available. Moreover, at some sampling dates, we were not able to find enough fresh dung pellet groups, so that we could not determine dung nutrient concentrations in some cases. The sampling size for each response variable at each sampling date is specified in the Online Resource Table S1.

From the sampling date- and site-specific average dung dry mass and the number of accumulated pellet groups per plot, we calculated the quantity of imported dung (kg ha^−1^) per plot for each sampling period. Multiplying the dung quantity with the nutrient concentrations returned the quantity of imported faecal N or P per plot for each sampling period. Similarly, multiplying the forage removal by red deer with the nutrient concentrations returned the quantity of exported N or P per plot for each sampling period. To account for changing nutrient concentrations in the course of a sampling period, we used the mean over nutrient concentrations at the start and end sampling date of a sampling period in all calculations. To avoid bias if one of the values at the start or end of a sampling period was missing, we filled in the mean over the existing values for the respective habitat type and sampling date. For the first sampling date (April 2015), plant nutrient concentrations were not available, so that in this case we employed the plant nutrient concentrations measured in April 2016.

Although it was not within the scope of our study to collect data on urine quantity and quality, we were interested in getting an estimate for the total amount of N imported by red deer through faeces and urine (*N*_total_). Consequently, we calculated the ratio of urinary to faecal N excretion (*U*_ratio_), according to the following equation (Mould and Robbins 1981; Hobbs 1996; c.f. Abbas et al. 2012):

$$U_{{{\text{ratio}}}} = \frac{{11.56 \times plant \, N + \frac{0.004}{{plant \, N \times w^{0.75} }} + 0.078}}{{0.05 + \frac{0.00421}{{plant \, N}}}}$$

with *w* = 100 kg as the approximate body mass of red deer. This equation enabled us to estimate the total *N* import (*N*_total_) by red deer:

$$N_{{{\text{total}}}} = faecal \,  N + U_{{{\text{ratio}}}} \times faecal \, N$$

As our study spanned a complete year, we obtained the annual rates of nutrient import or export (in kg ha^−1^) by summing up the mean values of observations per sampling period in heathlands and grasslands and derived the associated uncertainty by Gaussian error propagation.

To test for habitat-specific seasonal differences in nutrient concentrations and fluxes, we used linear mixed-effects models in the package nlme (Pinheiro et al. 2015). The habitat type (grasslands, heathlands) and the study period (five levels) and their interaction effect served as explanatory factors. We used a random intercept with treatment nested in sampling site to account for the spatial nestedness of the experimental design. In the models for faecal N and P concentrations, sampling site sufficed as random factor because we had collected the dung samples at site level.

For all models, we visually checked the normality and homogeneity of residuals and employed appropriate variance structure functions if needed to account for differences in variance between factor levels. To check for temporal autocorrelation between numeric sampling dates in our data set, we examined plots of autocorrelation estimates, but found that adding an autocorrelation structure to our models was not necessary. We used the AICc to compare model performance of all models nested in the global model using the dredge function in the MuMin package (Barton 2020) and report test statistics for the most parsimonious model. To test for differences between estimated marginal means of factor levels, we used the emmeans package (Lenth 2021).

In figures, we present boxplots of the raw data with the lower and upper hinges corresponding to the 25th and 75th percentiles. Outliers beyond the end of the whiskers, extending at most 1.5 of the distance between 25th and 75th percentiles, are plotted individually.

## Results

**Fig. 1:**
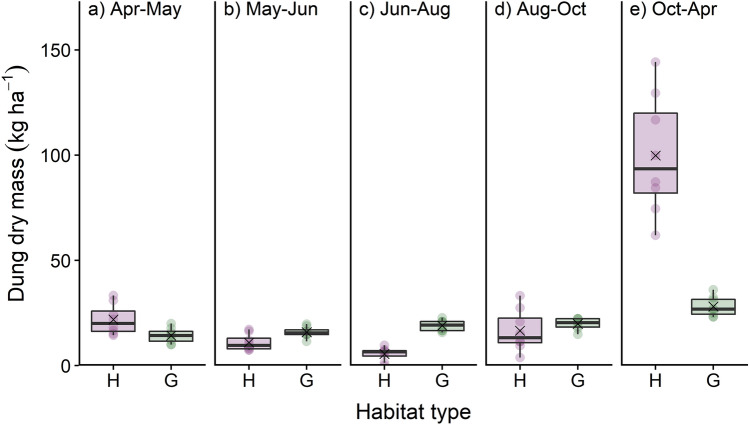
Seasonal variation between April 2015 and April 2016 of dung dry mass (kg ha^−1^) deposited by wild red deer in heathlands (H) and grasslands (G) in Grafenwöhr military training area, Germany. The cross symbol indicates the arithmetic mean; circles represent observations

We found between 0 and 112 dung pellet groups per plot and sampling date. Our decay experiment showed that the number of dung pellet groups observed per plot at each sampling date represented 85–100% of the actual number of dung pellet groups that had been deposited since the previous sampling date. On average, the corrected number of dung pellet groups per sampling plot and date was 21.5 ± 4.4 (mean ± SE) in heathlands, and 14.6 ± 1.1 in grasslands. This translated into an annually imported dung dry mass of 154.8 ± 14.6 kg ha^−1^ and 97.1 ± 2.7 kg ha^−1^ in heathlands and grasslands, respectively. In heathlands, the number of dung pellet groups and dung dry mass were higher in the winter than over the whole vegetation period. In grasslands, by contrast, the dung quantity accumulated over the course of the vegetation period was higher than in winter (Table 1; Fig. 1; Online Resource Tables S1, S2). Average plant N and P concentrations in the hand-pluck samples amounted to 1.307 ± 0.048% and 0.117 ± 0.005% in heathlands and 1.625 ± 0.093% and 0.216 ± 0.013% in grasslands, respectively (Online Resource Table S1), with significantly higher plant nutrient concentration in grasslands than in heathlands especially at the beginning of the vegetation period (Table 1, Online Resource Table S2).

**Table 1:** Effects of habitat type (heathlands, grasslands) and sampling date (May, Jun, Aug, Oct, Apr) or sampling period (Apr–May, May–Jun, Jun–Aug, Aug–Oct, Oct–Apr) on N and P concentrations in plant and faecal samples, import of dung dry matter, N and P import through red deer faeces, forage removal and N and P export by red deer grazing. Results of marginal Wald tests of the most parsimonious linear mixed effects model for each response variable. The conditional (*R*_(c)_^2^) and marginal (*R*_(m)_^2^) coefficients of determination express the variance explained by fixed and random effects combined and the variance explained only by fixed effects (Nakagawa et al. 2017)

Response	Model term	*df*_(num)_	*df*_(den)_	*F *value	*P *value	*R*_(m)_^2^	*R*_(c)_^2^
Plant N	Habitat type	1	6	12.81	0.012	0.94	0.99
	Sampling date	4	56	144.38	≤ 0.001		
	Habitat type × Sampling date	4	56	23.06	≤ 0.001		
Plant P	Habitat type	1	6	8.95	0.024	0.61	0.95
	Sampling date	4	56	42.58	≤ 0.001		
	Habitat type × Sampling date	4	56	9.45	≤ 0.001		
Faecal N	Habitat type	1	6	52.72	≤ 0.001	0.98	0.98
	Sampling date	4	21	31.45	≤ 0.001		
	Habitat type × Sampling date	4	21	74.82	≤ 0.001		
Faecal P	Habitat type	1	6	60.34	≤ 0.001	0.17	0.17
Dung dry matter import	Habitat type	1	6	7.98	0.030	0.98	0.99
	Period	4	56	52.76	≤ 0.001		
	Habitat type × Period	4	56	42.91	≤ 0.001		
N import	Habitat type	1	6	0.19	0.680	0.98	0.99
	Period	4	56	46.32	≤ 0.001		
	Habitat type × Period	4	56	25.06	≤ 0.001		
P import	Habitat type	1	6	259.37	≤ 0.001	0.99	0.99
	Period	4	58	47.59	≤ 0.001		
Forage removal	Habitat type	1	6	0.07	0.803	0.96	0.96
	Period	4	48	16.95	≤ 0.001		
	Habitat type × Period	4	48	14.22	≤ 0.001		
N export	Habitat type	1	6	0.52	0.497	0.79	0.79
	Period	4	48	10.60	≤ 0.001		
	Habitat type × Period	4	48	8.41	≤ 0.001		
P export	Habitat type	1	6	13.90	0.010	0.38	0.38
	Period	4	52	9.14	≤ 0.001

The concentration of N and P in red deer dung averaged 1.91 ± 0.09% and 0.24 ± 0.05% in heathlands and 2.73 ± 0.14% and 0.79 ± 0.06% in grasslands, respectively. While the observed faecal N concentration was significantly higher in grasslands than in heathlands only in May and June, the faecal P concentration was significantly higher in grasslands irrespective of sampling date (Table 1, Online Resource Tables S1, S2).

### Nutrient fluxes

**Fig. 2:**
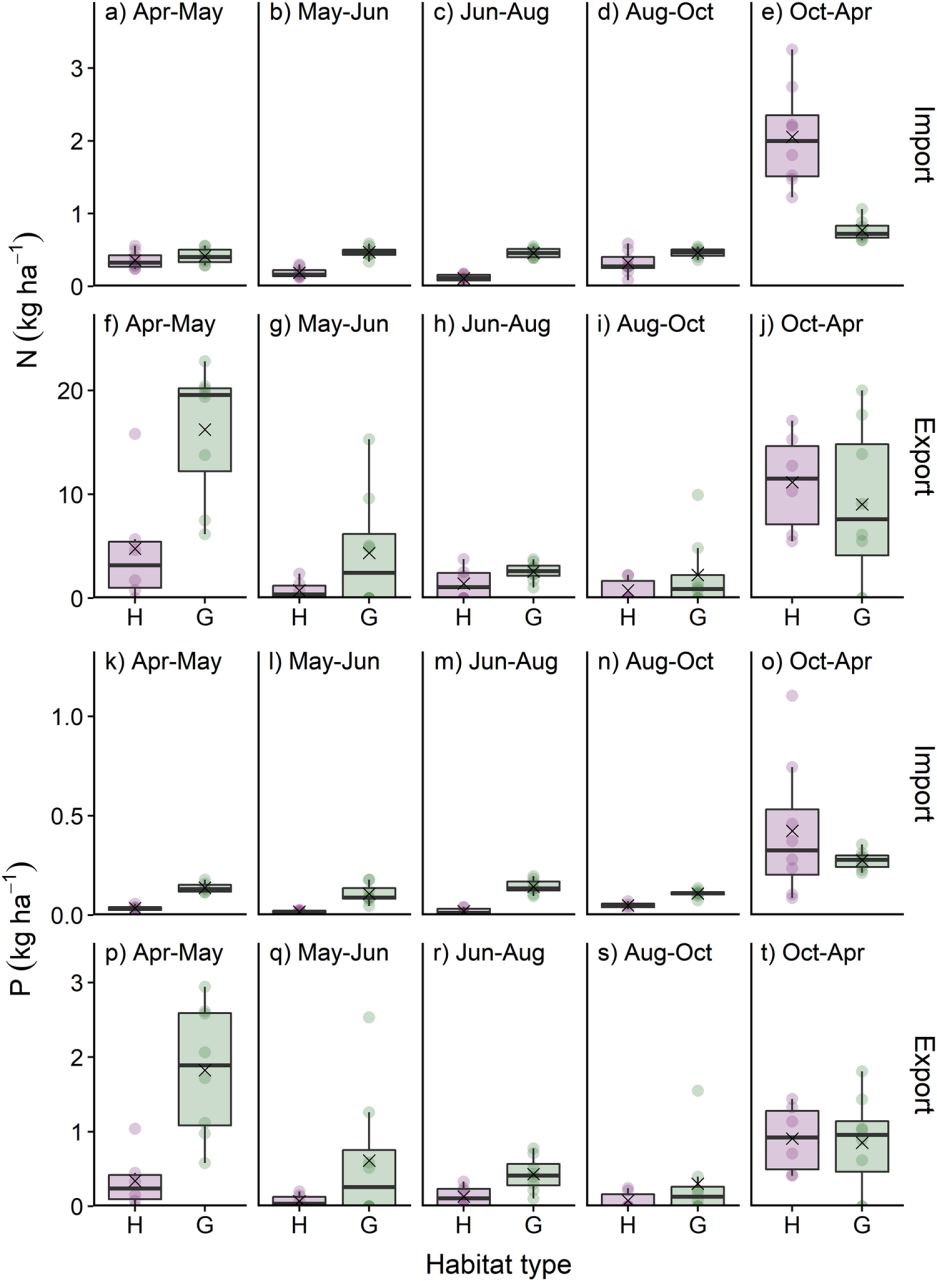
Seasonal import and export of **a**–**j** nitrogen (N) and **k**–**t** phosphorus (P) through defecation and grazing of wild red deer in heathlands (H) and grasslands (G) in Grafenwöhr military training area, Germany, from April 2015 to April 2016. The cross symbol indicates the arithmetic mean; circles represent observations

Defecation by red deer was associated with noticeable nutrient deposition, but the magnitude of nutrient import differed significantly between seasons and habitat types (Table 1; Fig. 2; Online Resource Tables S1, S2). In both heathlands and grasslands, the highest faecal import of N and P occurred in the winter period Oct–Apr, but the difference to the other periods was much more pronounced in heathlands. Except for the winter period, in which N import was higher in heathlands than in grasslands, N import was higher in grasslands, although the difference was not significant in the early spring period (Online Resource Table S2). The most parsimonious model for P import did not include the interaction between habitat type and sampling period, so that the estimated marginal means were higher in grasslands than in heathlands in all periods, although this did not reflect the raw data for P import being on average higher (but more variable) in heathlands in the winter period (Fig. 2o).

Forage removal by red deer showed habitat-specific seasonal patterns and was significantly higher in grasslands than in heathlands in Apr–May and Jun–Aug, while the opposite was true in the winter period (Online Resource Table S2). Grazing by red deer resulted in a substantial removal of N and P from the study plots (Fig. 2), which was in both habitats higher in the spring and winter period than in the three summer periods between May and October (Table 1; Online Resource Tables S1, S2). The N export in grasslands was significantly higher than in heathlands in the spring period Apr–May and in Jun–Aug (Online Resource Table S2). As the most parsimonious models for P export did not include an interaction term between habitat type and study period, estimated marginal means for P export were generally higher in grasslands than in heathlands, whereas the raw data suggested that P export in winter was rather similar between habitat types (Table 1; Online Resource Table S2, Fig. 2t).

On an annual basis, the nutrient export by red deer exceeded the nutrient import in both heathlands and grasslands (Table 2). The difference between N import and N export remained negative even when accounting for the total N import including the N fraction excreted via urine (approximated using the ratio of urinary to faecal N excretion, i.e., 0.65 ± 0.03 in heathlands and 0.92 ± 0.06 in grasslands (Online Resource Table S1)). The difference between annual import and export of both N and P was much more pronounced in grasslands than in heathlands, the 95% confidence intervals overlapped only slightly between habitats (Table 2). The net nutrient removal amounted to approximately 40–50% of the N and 30–40% of the P in the annually produced aboveground plant biomass (Online Resource Table S3).

**Table 2:** Annual import and export and their difference (import–export) of nitrogen (N) and phosphorus (P) in kg ha^−1^ a^−1^ through excretion and grazing of wild red deer in heathlands and grasslands in Grafenwöhr military training area, Germany. The total N import represents the sum of faecal N import and estimated urinary N import. The bracketed numbers give the lower and upper limit of the 95% confidence interval

	Faecal N import	Total N import	N export	∆N	P import	P export	∆P
Heathlands	3.03 [2.32, 3.74]	4.91 [3.81, 6.01]	18.81 [9.91, 27.71]	− 13.90 [− 22.87, − 4.93]	0.44 [0.24, 0.64]	1.52 [0.81, 2.23]	− 1.08 [− 1.82, − 0.34]
Grasslands	2.58 [2.36, 2.79]	4.92 [4.48, 5.36]	34.41 [22.69, 46.14]	− 29.50 [− 41.23, − 17.76]	0.73 [0.66, 0.81]	4.02 [2.54, 5.51]	− 3.29 [− 4.78, − 1.80]

## Discussion

For two protected semi-natural habitat types in an area inhabited by a large population of free-ranging red deer, we showed that the annual nutrient export by grazing was markedly higher than the excremental import of nutrients. We thus provided support for our hypothesis H1a that wild red deer can counteract atmospheric nutrient deposition in semi-natural open habitats because nutrient removal by grazing was high and import through excreta was low. This finding is promising for habitat conservation, as nutrient enrichment poses a major threat to many protected habitats (EEA 2020). Our study hence supports the view that integrating wild red deer into the management of open landscapes can be a suitable and promising conservation strategy, similar to extensive livestock grazing (Van Wieren 1995; Rosenthal et al. 2012), which is usually achieved by domestic cattle (Pykälä 2003), sheep (Pakeman et al. 2003), goats (Elias and Tischew 2016) or multi-species assemblages (Loucougaray et al. 2004; Henning et al. 2017; Fraser and Rosa García 2018), and has been shown to counteract atmospheric nutrient deposition in different open habitat types (Kooijman and Smit 2001; Uytvanck et al. 2010).

### Magnitude of nutrient import and export by red deer

Nutrient dynamics by free-ranging herbivores in semi-natural open habitats have been quantified in few cases (e.g., Schoenecker et al. 2004; Schütz et al. 2006). Large variations in environmental conditions, vegetation composition and productivity, herbivore species, population density and management need to be considered when comparing absolute values of nutrient import and export by herbivores between studies. With higher herbivore abundance and higher diet quality, naturally higher nutrient fluxes though herbivores can be expected, but for habitat conservation, the balance between nutrient import and export needs to be considered.

For wild red deer in the Rocky Mountains, Colorado, USA, Schoenecker et al. (2004) reported an average dung deposition of 346 and 605 kg ha^−1^ a^−1^ in mesic meadows and upland grass/shrub vegetation, resulting in a total excremental N deposition (faeces and urine) of 10.5 and 18.3 kg ha^−1^ a^−1^, respectively, which is more than twice the average total N deposition by red deer in our study. Similarly, in a Belgian conservation area grazed by cattle stocked at 0.2 animal units ha^−1^ a^−1^, N import in grasslands, wooded pastures and unvegetated sites (ca. 15–25 kg N ha^−1^ a^−1^; Uytvanck et al. 2010) was higher than in our study; however, the lowest N import, recorded in forested habitat, was comparable to the total N import we observed for wild red deer in open habitats.

N export by grazing in the study of red deer in the Rocky Mountains was estimated at 26.9 and 5.2 kg ha^−1^ a^−1^ in mesic meadows and upland grass/shrub vegetation, which was less than the average N export in grasslands and heathlands in our study (Schoenecker et al. 2004). Still, similarly to our study, the difference between N import and export in mesic meadows in the Rocky Mountains was negative. By contrast, a net N gain through red deer excreta was observed in upland grass/shrub vegetation (Schoenecker et al. 2004). In the Belgian conservation area, cattle grazing removed about 20 kg N ha^−1^ a^−1^ from both grassland and wooded pasture, mainly during spring and summer (Uytvanck et al. 2010), while N export in forest was much lower and occurred only in winter, which is reminiscent of the seasonal patterns of N export by red deer we observed in grasslands vs. heathlands.

The annual P export in both habitat types of our study was higher than the P removal by red deer in a subalpine grassland area of the Swiss National Park, which averaged 0.95 and 0.49 kg P ha^−1^ a^−1^ in short- and tall-grass vegetation, respectively (Schütz et al. 2006). Annual P import through red deer faeces in that study ranged from 0.01 to 1.58 kg P ha^−1^ a^−1^, which is notably higher than the P import in our study. This is related to the higher imported dung quantity ranging between 3.5 and 400 kg ha^−1^ in the Swiss National Park at an average faecal P concentration closer to the faecal P concentration in heathlands than in grasslands of our study (0.39%; Schütz et al. 2006).

These results from different studies illustrate that differences between habitat types are crucial for the balance between nutrient import and export by large herbivores. Based on the higher productivity and forage quality and resulting higher attractiveness of grasslands as a foraging habitat for red deer (Riesch et al. 2019), we hypothesized that the nutrient export in this habitat would be higher and the difference between nutrient export and nutrient import would be larger than in heathlands (H2a). The N import by faeces and urine of red deer was similar in both habitat types, although the overall dung quantity deposited by red deer in heathlands was higher (but more variable) than in grasslands. By contrast, the annual P import was lower in heathlands owing to lower faecal P concentrations. Combined with the higher nutrient export in grasslands than in heathlands resulting from seasonally elevated forage removal and plant nutrient concentrations, this led to a more negative difference between import and export of nutrients in grasslands. This result is important when considering the implications for habitat conservation in view of atmospheric nutrient deposition.

### Implications for habitat conservation in view of atmospheric nutrient deposition

Critical load ranges defined at the European level are supposed to prevent negative effects of atmospheric N deposition on biodiversity and ecosystem functioning in habitat types with different sensitivity to increased N availability (Bobbink et al. 2015). For dry inland heaths, the established critical N load is 10–20 kg N ha^−1^ a^−1^. For hay meadows, in which biomass and nutrients are regularly removed by mowing, the critical load range is higher (20–30 kg N ha^−1^ a^−1^; Bobbink et al. 2015). However, for our heathland habitat type 4030, an observational study from Ireland showed that critical changes in plant species abundances and species loss can already be expected if the annual N deposition exceeds 4.1 kg ha^−1^. For the grassland habitat type 6510, significant changes in plant community composition occurred already at > 7.5 kg N ha^−1^ a^−1^ (Wilkins et al. 2016). As a consequence, the annual background deposition of 9–11 kg N ha^−1^ a^−1^, derived for our study area from model results (Umweltbundesamt 2021), could potentially impact on the characteristic plant communities in our study. However, even when considering the conservative lower limit of the 95% confidence interval, the net N export by red deer in the GTA should be able to prevent grasslands from receiving detrimental N input at current atmospheric deposition levels. For heathlands, this could not be completely excluded, as weighing up the smallest expectable net N export by red deer according to the 95% confidence interval against an atmospheric deposition of 11 kg N ha^−1^ a^−1^ returns a possible net input of ca. 6 kg N ha^−1^ a^−1^. As, however, an earlier study on plant diversity and soil nutrient conditions in the GTA found that neither grassland nor heathland plant communities showed evidence of nutrient enrichment (Riesch et al. 2018), heavy nutrient input during the last decades seems unlikely.

At a scale beyond our experimental area, N deposition rates vary considerably, e.g., between < 10 and > 25 kg N ha^−1^ a^−1^ in Germany (Umweltbundesamt 2021) and, despite decreasing trends in the past decades, can reach more than 30 kg N ha^−1^ a^−1^ in some European regions (Strien et al. 2018; Dirnböck et al. 2018). Even at higher atmospheric deposition, our results suggest that a grassland area with the same level of red deer grazing as in GTA would in most cases receive no or only minor net N import due to the high N export by red deer. As heathlands are more sensitive to N deposition and the N export in heathlands was lower, we assume that at higher atmospheric deposition levels, red deer grazing can mitigate atmospheric N deposition, but might not always prevent a potentially harmful net N import in heathlands.

Atmospheric P deposition in Europe is estimated at 0.3 kg P ha^−1^ a^−1^ (Tipping et al. 2014). Under such a P deposition level, red deer in GTA would still ensure a small (heathlands) or considerable (grasslands) net P depletion. Even when considering that P deposition at regional scale can be much higher, e.g., related to intensive agricultural production (Tipping et al. 2014), it still seems very unlikely that similar grasslands with a grazing pressure by red deer as observed in our study would receive a net import of P. This is a very important finding, which might help to explain why red deer grazing is beneficial for plant diversity in this habitat type (Riesch et al. 2020), as increases in plant-available P are related to decreasing plant diversity in grasslands (Ceulemans et al. 2014; Riesch et al. 2018). For heathlands, however, a net P import might not be ruled out despite red deer grazing if atmospheric P deposition was higher than average.

### Faecal nutrient concentrations

The observed faecal N concentrations of red deer in our study were lower in heathlands than in grasslands, in which faecal N concentrations covered a similar range as known from red deer in Mediterranean habitats in Spain during spring time (1.7–3.4% N; Carpio et al. 2015). The average faecal N concentrations of red deer in the fenced Dutch nature reserve Oostvaardersplassen (1.62% N in winter, 2.58% N in spring; Valdés-Correcher et al. 2019) were closer to the faecal N concentrations in heathlands than in grasslands of our study. Compared to faecal N of red deer in Montana, USA, averaging 1.46% N during a time of nutritional deprivation in winter (Christianson and Creel 2007), the faecal N concentrations were higher in both heathlands and grasslands throughout the year, suggesting that animals in our study area ingested forage with better nutrient availability or digestibility.

The faecal P concentration of red deer in GTA differed strongly between grasslands and heathlands, mirroring the higher P concentration in grassland vegetation, which potentially relates to the difference in plant-available soil P between the two habitat types (Riesch et al. 2018). In comparison to the faecal P concentrations we observed in heathlands and grasslands, red deer had intermediate faecal P concentrations in Oostvaardersplassen (0.30% and 0.53% in winter and spring; Valdés-Correcher et al. 2019). The differences in faecal N and P concentrations between heathlands and grasslands translated into a higher average dung N:P ratio in heathlands than in grasslands (Online Resource Table S1, Fig. S2), which conforms to the higher dung N:P ratio in browsing than in grazing animals (Sitters and Andriuzzi 2019; Valdés-Correcher et al. 2019). This might be important for the diversity in plant communities, as there is experimental evidence that the dung N:P ratio can affect plant competitive dynamics, i.e. dung with high N:P ratio promoted grasses, while dung with lower N:P ratio benefited N-fixing legumes, which have higher relative P requirements (Valdés-Correcher et al. 2019). It has been hypothesized that the relative amount of N and P released in excreta of herbivores could even affect the nutrient limitation status of plant communities (Sitters et al. 2017). In our study, average plant N:P ratios close to or lower than 10 (11.33 ± 0.34 and 8.15 ± 0.45 in heathlands and grasslands) indicated N- rather than P-limitation of plant growth, especially in grasslands (Güsewell 2004). That the plant N:P ratio of heathlands in GTA is low compared to reported values from this habitat type at other places in Europe might be a consequence of the historically lacking agricultural inputs in the military training area (Riesch et al. 2018) and of occasional fires due to military activities, which remove relatively more N than P (Roem and Berendse 2000). Low or intermediate plant N:P ratios in heathlands are regarded as favourable for the preservation of plant species (Roem and Berendse 2000) and biodiversity at higher trophic levels (Vogels et al. 2013, 2017). Although the N:P ratio of red deer excreta in heathlands tended to be higher than the plant N:P ratio, and in grasslands, the N:P ratio in red deer dung was lower than in the vegetation (Online Resource Fig. S2), we do not expect red deer to have a substantial effect on habitat nutrient limitation status, as the ratio of net N to net P export (12.9 in heathlands and 9.0 in grasslands) was remarkably similar to the plant N:P ratio in both habitat types. At the microsite scale, however, we might speculate that spots receiving red deer dung in grasslands could provide favourable conditions for P-demanding legumes and thus contribute to promote biodiversity in this grass-dominated habitat type (Riesch et al. 2018). In contrast, spots receiving urine could benefit plants with higher N requirements, such as grasses, or might result in small gaps through urine scorching (Whitehead 2000; Bokdam and Gleichman 2000).

## Limitations

Our study aimed at weighing the nutrient import through herbivore excreta against the export through grazing, which represent only part of the nutrient balance. We thus did not account for nutrient losses from urine and dung by processes such as leaching, ammonia volatilization, denitrification and nitrous oxide emissions, erosion and surface runoff (Dahlin et al. 2005; Laubach et al. 2013; Zhou et al. 2017b), neither did we account for N volatilization from senescing plants (Hobbs 1996). The amount of N we measured in red deer faeces might not equal the effective amount of N reaching the soil system because of gaseous losses that occur after defecation. As we intended to collect fresh dung and volatilisation from dung might become significant only after several days (Laubach et al. 2013), our measured faecal N might include a portion of N that still would have volatilized when the dung had remained in the field. The proportion of faecal N lost through ammonia volatilization, however, is often much less than 10% of the N deposited (Ryden et al. 1987; Petersen et al. 1998) and the percentage of nitrous oxide losses from dung is even lower (Wachendorf et al. 2008). Additionally, we cannot exclude that during the time between defecation and dung sample collection some N as well as P might have leached from dung to soil, reducing the nutrient concentrations in our samples. As the release of P from dung is more strongly related to the physical decomposition of the dung than to leaching (Haynes and Williams 1993; Aarons 2004), P losses should have been minimal from our seemingly fresh dung samples. The small soluble fraction of faecal N might leach quickly by rain, but mineralisation of N from dung is generally slow (Whitehead 2000) and thus should not have changed the N concentration in our samples considerably. For urinary N deposited by ruminants on pasture in agricultural systems, it is assumed that typically 13% is volatilized as NH_3_, 2% is emitted as NO_x_ and 20% leached as nitrate, whereby these processes depend on soil and urine characteristics, weather and environmental conditions (Selbie et al. 2015). Such processes reducing the effective amount of N from urine a system receives are not reflected by our approximation of urinary N based on the N measured in red deer faeces. Regarding nutrient imports, we did not account for the effects of biological N_2_-fixation, neither for carcasses nor shed antlers (Flueck 2009). Furthermore, we did not consider the occurrence of more complex interactions, such as trampling-mediated changes in plant root morphology and functioning, which might, in turn, moderate soil nutrient availability (Sitters et al. 2017; Yu et al. 2021).

In addition, we point out that the precision of our calculations of nutrient fluxes is somewhat limited as we collected data under field conditions. Measuring the urine quantity released per plot and assessing the urinary N concentration directly was thus not possible. Instead, we calculated the ratio of urinary to faecal N excretion based on an approximated average red deer body weight and the plant N concentration in hand-pluck samples, which might not exactly equal the N concentration in the actual red deer diet. Besides, we assumed that spatial patterns of urine release would not deviate from defecation patterns. However, given the markedly negative difference between import and export of both N and P by red deer, we are confident that our results are qualitatively robust, providing evidence that red deer grazing leads to a net depletion of N and P, especially in the studied lowland hay meadows. This result is all the more remarkable as comparing the observed faecal N concentration with the expected faecal N concentration calculated from the plant N concentration in our hand-pluck samples gave us reason to believe that our study plots might have received red deer dung at least partially produced from vegetation with higher nutrient concentrations than in the forage growing on our study plots (Online Resource Appendix A1, Fig. S3).

While we demonstrated that the two studied open habitat plant communities benefited from a net removal of nutrients through red deer grazing, our data set from permanent plots was not suitable for tracking the flow of nutrients transported by red deer in our study area. As the grazing animals absorb only a small fraction of the N and P ingested (usually 5–15%) and nutrient concentrations in body tissue are low (Whitehead 2000), the nutrient export from the system with harvested animals is small (ca. 0.13 kg N ha^−1^a^−1^ and 0.05 kg P ha^−1^a^−1^ based on the weight of red deer culled in the hunting districts of our sampling sites; Online Resource Table S4) compared to the internal nutrient fluxes through forage removal and excretion of red deer. Hence, we expect that there is a substantial lateral transport of nutrients related to the spatio-temporal habitat selection patterns of red deer in our study area (Richter et al. 2020). Accumulation and depletion zones of N and P are well known in livestock grazing systems (Jewell et al. 2007; Schnyder et al. 2010; Koch et al. 2018). Also for free-ranging red deer in the Rocky Mountains, Schoenecker et al. (2004) suggested that the animals enrich nutrients in resting sites, while depleting nutrients in areas mainly used for foraging. Consequently, to fully understand the nutrient dynamics mediated by red deer and their differential effects in different habitat types, studies at a larger spatial scale are required to identify potential areas of nutrient accumulation through excreta. Furthermore, data on plant and soil nutrient concentrations from long-term exclosures would be highly valuable to evaluate how the nutrient fluxes through red deer affect soil nutrient availability for plant communities.

## Conclusion

By quantifying the magnitude of nutrient import and export by red deer, our study contributes to a more holistic understanding of the effects of wild red deer in semi-natural open habitats. Given a relatively high red deer density (compared to common population target levels in forestry) and a wildlife management regime warranting that red deer feel safe foraging in the open landscape, grazing by wild red deer can mitigate if not compensate for current levels of atmospheric nutrient deposition in different open habitat types, similarly to extensive livestock grazing (Kooijman and Smit 2001; Uytvanck et al. 2010). Thus, our results confirm that red deer grazing is not only an appropriate management option for open habitats in terms of biomass removal (Riesch et al. 2019), effects on vegetation structure and diversity (Riesch et al. 2020) or seed dispersal (Iravani et al. 2011; Wichelhaus 2020), but also regarding nutrient dynamics. Accordingly, we support the idea of red deer grazing as an additional tool for nature conservation management and see high potential for large target areas in which conventional management is not feasible.

## Supplementary Information

Below is the link to the electronic supplementary material.

Supplementary file1 (PDF 735 KB)

## Data Availability

The data were deposited in the Zenodo open-access repository and can be accessed at https://doi.org/10.5281/zenodo.5554935.

## Code availability

The R code used to analyse data in the current study is available from the corresponding author on reasonable request.
