# Supplementary material for: Grazing by wild red deer can mitigate nutrient enrichment in protected semi-natural open habitats
Source: Oecologia. 2022 May 12;199(2):471–85. doi: 10.1007/s00442-022-05182-z (PMC9225971; doi:10.1007/s00442-022-05182-z)
Supplement: Supplementary file 1 — Supplementary file1 (PDF 735 KB) [file 442_2022_5182_MOESM1_ESM.pdf]

Supplementary Information

to the paper Riesch et al. (2022) Grazing by wild red deer can mitigate nutrient enrichment in protected semi-natural open habitats. Oecologia. https://doi.org/10.1007/s00442-022-05182-z

Corresponding author email: friederike.riesch@agr.uni-goettingen.de

Appendices

Appendix A1 Faecal N concentrations observed in red deer dung and predicted based on plant N concentration

Tables

Table S1 Summary values per habitat type and sampling date of all variables of interest used in the present study

Table S2 Estimated marginal means from linear mixed effects models

Table S3 Amount of nitrogen and phosphorus in the aboveground net primary productivity

Table S4 Approximation of annual nutrient export through culled red deer

Table S5 Marginal Wald test results for the linear mixed effects model analysing differences between observed and expected faecal N concentration

Table S6 Estimated marginal means from the linear mixed effects model for observed vs. expected faecal N concentration

Figures

Fig. S1 Map of the study area

Fig. S2 Nitrogen to phosphorus ratio in dung and excreta of red deer and in vegetation

Fig. S3 Observed and expected faecal N concentration of red deer

References

Appendix A1 Faecal N concentrations observed in red deer dung and predicted based on plant N concentration

To evaluate if the diet consumed by red deer was of similar quality as the vegetation on our experimental plots, we additionally predicted the expected faecal N percentage (N_{exp}) based on the plant N concentration in our hand-pluck samples as follows (Moen and DelGiudice 1997):

$$N_{exp} = 0.941 + 0.479 * plant\ N$$

Then we calculated the ratio of observed faecal N concentration to expected faecal N concentration (Christianson and Creel 2010).

We explored differences between the observed and expected N concentrations in red deer dung using N concentration as response and data type (observed/expected) as well habitat type (grassland/heathland) and sampling date (five levels) and all interactions as explanatory variables. As we had collected the dung samples at site level, the expected faecal N concentrations calculated from hand-pluck samples for each of the two plots per sampling site were averaged. Accordingly, sampling site sufficed as random factor.

In heathlands, the faecal N concentration predicted based on plant N concentration in hand pluck samples was significantly lower than the observed faecal N concentration except for the winter period Oct–Apr, in which the difference was not significant, and the spring period Apr–May, in which the expected N was higher than the observed N (Online Resource Fig. S3, Tables S5, S6). In grasslands, the expected faecal N concentration was significantly lower than the observed N concentration in red deer dung for all sampling dates. The ratio of observed:expected faecal N averaged per period ranged between 0.92 and 1.43 in heathlands and between 1.53 and 1.72 in grasslands (Online Resource Table S1).

From the difference between observed faecal N and faecal N predicted based on plant N we deduce that the diet red deer actually consumed often had a better forage quality than the vegetation we cut on our plots when collecting the hand-pluck samples. The ratio of observed:expected faecal N was higher than 1.5 in grasslands throughout the year, but rather close to one in heathlands except for the summer periods. As red deer are intermediate feeders (Hofmann 1989), able to switch between bulk feeding and concentrate selecting strategies, it is likely that they did not take bites randomly as we did when collecting the hand-pluck samples at plot-scale, but grazed more selectively choosing plants with elevated N concentration (Langvatn and Hanley 1993; Semiadi et al. 1995). Especially female red deer are highly selective in foraging, mostly from late winter until parturition (Čupić et al. 2021). The generally more balanced ratio of observed:expected faecal N in heathlands probably resulted from a lower availability of plants with high N concentration in this habitat, which is characterized by extremely low soil nutrient levels (Riesch et al. 2018). That the ratio of observed:expected faecal N

concentration in heathlands was higher during summer than during the rest of the year could be a result of red deer feeding in other habitats in that time of the year, which is likely, as significant red deer forage removal occurred in heathlands only during the winter period (Riesch et al. 2019). Our main quantitative result that the nutrient export mediated by red deer exceeded the import in both habitat types gains even more relevance under the assumption that study plots might have received red deer dung at least partially produced from vegetation with higher nutrient concentrations growing at other places.

We are, however, aware that our comparison of observed to expected faecal N is based on rather rough estimates, as we used a regression for predicting faecal N from plant N derived from a limited data set (n = 58, r^2 = 0.64 (Moen and DelGiudice 1997)) comprising different ruminant species. In addition, faecal N might actually be higher than predicted when animals feed on vegetation high in secondary plant compounds, such as tannins and other phenolics (Moen and DelGiudice 1997). The N concentration in dung is usually interpreted as a proxy for diet digestibility, but when secondary plant compounds reduce the digestibility of dietary N while increasing faecal N concentration, this might not be valid (Leslie et al. 2008; Arturo et al. 2015). Secondary plant compounds are supposed to be low or absent in a grass-based diet (Gordon 2003; Verheyden-Tixier et al. 2008), whereas ericaceous plants contain substantial concentrations of tannins and phenolics (Hofland-Zijlstra and Berendse 2009). Because of the potential occurrence of secondary plant compounds, we suppose that the expected faecal N values in heathlands, but not so much in grasslands, might represent underestimates and the measured faecal N values might indicate a higher digestibility than actually present. Furthermore, gastrointestinal parasites damaging the digestive tract can increase the faecal N concentration independently from diet N due to increased metabolic N losses (Arturo et al. 2015). Additionally, irrespective of diet N, animals are able to extract a higher fraction of N in winter, leading to lower faecal N concentrations (Arnold et al. 2015), which might have contributed to the closer observed:expected faecal N ratio in heathlands in winter. While these considerations do not compromise our quantitative assessment of nutrient fluxes and stoichiometry, we are hence cautious with inferences regarding the digestibility of red deer diets in our study system.

Table S1 Sample size (*n*), mean values, standard deviation (SD) and standard error (SE) of all variables of interest used in the present study per habitat type and sampling date/period (the sampling dates from May to October were performed in the year 2015, while the final sampling date was in April 2016)

		Heathlands				Grasslands			
	Sampling date/period	*n*	Mean	SD	SE	*n*	Mean	SD	SE
Dung pellet groups (ha^{-1})	May	8	641.67	211.30	74.71	8	467.84	116.62	41.23
	Jun	8	336.18	118.59	41.93	8	506.54	82.60	29.20
	Aug	8	177.78	104.23	36.85	8	537.04	75.53	26.70
	Oct	8	388.89	231.55	81.87	8	513.51	69.08	24.42
	Apr	8	3233.62	916.91	324.18	8	1219.30	203.71	72.02
Dung dry mass (ha^{-1})	May	8	21.87	7.20	2.55	8	14.30	3.56	1.26
	Jun	8	10.98	3.87	1.37	8	15.89	2.59	0.92
	Aug	8	5.54	3.25	1.15	8	19.02	2.68	0.95
	Oct	8	16.60	9.88	3.49	8	19.84	2.67	0.94
	Apr	8	99.83	28.31	10.01	8	28.06	4.69	1.66
Vegetation productivity (kg ha^{-1})	Apr–May	6	-96.95	1842.37	752.15	8	1493.64	505.88	178.85
	May–Jun	6	-163.47	929.97	379.66	8	1922.79	975.55	344.91
	Jun–Aug	6	495.94	960.00	391.92	8	271.80	227.79	80.54
	Aug–Oct	6	58.49	984.00	401.71	8	-853.08	651.06	230.18
	Oct–Apr	6	-22.29	1152.33	470.44	8	-165.02	1411.59	499.07
Forage removal (kg ha^{-1})	Apr–May	6	300.18	347.05	141.68	8	717.95	296.67	104.89

		Heathlands				Grasslands			
	Sampling date/period	*n*	Mean	SD	SE	*n*	Mean	SD	SE
	May–Jun	6	49.91	65.67	26.81	8	243.31	317.74	112.34
	Jun–Aug	6	115.41	132.63	54.15	8	206.45	61.10	21.60
	Aug–Oct	6	72.63	116.28	47.47	8	214.10	342.34	121.04
	Oct–Apr	6	949.50	402.35	164.26	8	531.01	430.86	152.33
Plant N concentration (%)	May	8	1.66	0.21	0.08	8	2.26	0.11	0.04
	Jun	8	1.40	0.15	0.05	8	1.36	0.12	0.04
	Aug	8	1.01	0.13	0.05	8	1.09	0.15	0.05
	Oct	8	1.03	0.15	0.05	8	1.09	0.08	0.03
	Apr	8	1.45	0.21	0.07	8	2.33	0.33	0.12
N export (kg ha^{-1})	Apr–May	6	4.77	5.84	2.38	8	16.23	6.34	2.24
	May–Jun	6	0.75	0.97	0.40	8	4.35	5.65	2.00
	Jun–Aug	6	1.40	1.63	0.66	8	2.57	0.91	0.32
	Aug–Oct	6	0.74	1.15	0.47	8	2.24	3.49	1.23
	Oct–Apr	6	11.15	4.76	1.94	8	9.03	7.58	2.68
Observed faecal N concentration (%)	May	3	1.60	0.09	0.05	3	3.49	0.05	0.03
	Jun	4	1.79	0.07	0.03	4	2.43	0.08	0.04
	Aug	3	2.04	0.51	0.29	4	2.39	0.18	0.09
	Oct	4	2.05	0.25	0.12	4	2.27	0.18	0.09

		Heathlands				Grasslands			
	Sampling date/period	*n*	Mean	SD	SE	*n*	Mean	SD	SE
Expected faecal N concentration (%)	Apr	4	2.05	0.61	0.30	4	3.25	0.73	0.36
	May	8	1.73	0.10	0.04	8	2.02	0.05	0.02
	Jun	8	1.61	0.07	0.03	8	1.59	0.06	0.02
	Aug	8	1.42	0.06	0.02	8	1.46	0.07	0.03
	Oct	8	1.43	0.07	0.03	8	1.46	0.04	0.01
Observed:expected faecal N ratio	Apr	8	1.64	0.10	0.03	8	2.06	0.16	0.06
	May	6	0.92	0.03	0.01	6	1.72	0.05	0.02
	Jun	8	1.11	0.05	0.02	8	1.53	0.04	0.01
	Aug	6	1.43	0.38	0.15	8	1.64	0.18	0.06
	Oct	8	1.43	0.18	0.06	8	1.55	0.13	0.05
Urinary:faecal N ratio	Apr	8	1.27	0.40	0.14	8	1.60	0.40	0.14
	May	8	0.83	0.10	0.04	8	1.50	0.17	0.06
	Jun	8	0.81	0.14	0.05	8	1.04	0.07	0.02
	Aug	8	0.57	0.09	0.03	8	0.59	0.08	0.03
	Oct	8	0.45	0.09	0.03	8	0.50	0.06	0.02
Faecal N import (kg ha^{-1})	Apr	8	0.60	0.11	0.04	8	0.96	0.14	0.05
	Apr–May	6	0.36	0.12	0.04	6	0.42	0.11	0.04
	May–Jun	8	0.19	0.07	0.02	8	0.47	0.08	0.03

		Heathlands				Grasslands			
	Sampling date/period	*n*	Mean	SD	SE	*n*	Mean	SD	SE
	Jun–Aug	6	0.11	0.06	0.02	8	0.46	0.07	0.02
	Aug–Oct	8	0.32	0.16	0.06	8	0.46	0.06	0.02
	Oct–Apr	8	2.05	0.69	0.24	8	0.77	0.15	0.05
Total N import (kg ha^{-1})	Apr–May	6	0.66	0.23	0.08	6	1.04	0.29	0.10
	May–Jun	8	0.34	0.15	0.05	8	0.96	0.17	0.06
	Jun–Aug	6	0.17	0.10	0.04	8	0.73	0.10	0.04
	Aug–Oct	8	0.47	0.26	0.09	8	0.69	0.10	0.03
	Oct–Apr	8	3.26	1.05	0.37	8	1.50	0.25	0.09
Plant P concentration (%)	May	8	0.15	0.03	0.01	8	0.31	0.07	0.03
	Jun	8	0.13	0.03	0.01	8	0.21	0.06	0.02
	Aug	8	0.10	0.01	0.00	8	0.19	0.10	0.03
	Oct	8	0.10	0.02	0.01	8	0.17	0.07	0.02
	Apr	8	0.10	0.03	0.01	8	0.20	0.05	0.02
P export (kg ha^{-1})	Apr–May	6	0.34	0.38	0.16	8	1.82	0.87	0.31
	May–Jun	6	0.07	0.09	0.04	8	0.61	0.90	0.32
	Jun–Aug	6	0.13	0.15	0.06	8	0.43	0.23	0.08
	Aug–Oct	6	0.08	0.12	0.05	8	0.30	0.52	0.19
	Oct–Apr	6	0.91	0.46	0.19	8	0.85	0.64	0.22

		Heathlands				Grasslands			
	Sampling date/period	*n*	Mean	SD	SE	*n*	Mean	SD	SE
Faecal P concentration (%)	May	3	0.15	0.03	0.02	3	1.04	0.13	0.08
	Jun	4	0.16	0.04	0.02	4	0.67	0.30	0.15
	Aug	3	0.27	0.23	0.13	4	0.75	0.18	0.09
	Oct	2	0.20	0.01	0.01	4	0.55	0.07	0.03
	Apr	4	0.40	0.31	0.16	4	0.99	0.11	0.06
P import (kg ha^{-1})	Apr–May	6	0.038	0.013	0.005	6	0.120	0.026	0.009
	May–Jun	8	0.017	0.006	0.002	8	0.137	0.038	0.013
	Jun–Aug	6	0.012	0.009	0.003	8	0.135	0.043	0.015
	Aug–Oct	4	0.043	0.011	0.005	8	0.129	0.027	0.010
	Oct–Apr	8	0.328	0.201	0.071	8	0.213	0.028	0.010
Plant N:P ratio	May	8	11.12	1.64	0.58	8	7.66	1.63	0.58
	Jun	8	10.92	1.34	0.47	8	7.03	1.98	0.70
	Aug	8	9.85	0.67	0.24	8	7.05	3.75	1.33
	Oct	8	10.40	1.27	0.45	8	7.01	2.47	0.87
	Apr	8	14.37	2.22	0.78	8	12.02	2.38	0.84
Dung N:P ratio	May	3	11.22	2.39	1.38	3	3.38	0.51	0.29
	Jun	4	11.41	2.55	1.28	4	4.10	1.49	0.74
	Aug	3	10.13	4.55	2.63	4	3.31	0.78	0.39

		Heathlands				Grasslands			
	Sampling date/period	*n*	Mean	SD	SE	*n*	Mean	SD	SE
Excreta N:P ratio	Oct	2	9.35	1.34	0.95	4	4.17	0.35	0.18
	Apr	4	6.83	3.42	1.71	4	3.28	0.47	0.24
	May	3	21.28	6.47	3.74	3	8.21	1.43	0.83
	Jun	4	19.59	5.18	2.59	4	6.89	2.35	1.17
	Aug	3	14.77	6.91	3.99	4	4.84	1.03	0.51
	Oct	2	13.76	2.30	1.62	4	6.29	0.38	0.19
	Apr	4	13.04	7.75	3.87	4	8.90	1.09	0.55

Table S2 Estimated marginal means and related standard errors (SE), degrees of freedom (*df*) and 95%-confidence limits (CL) from linear mixed effects models for observed plant and faecal concentrations of N and P, the quantity of dung deposited by red deer, N and P import through red deer faeces as well as N and P export by red deer grazing. *P*-values are associated with the contrasts between habitats within period. *P*-values <0.05 are printed in bold

Response	Habitat type	Sampling date/period	Estimate	SE	*df*	Lower CL	Upper CL	*P*-value
Plant N (%)	Heathlands	May	1.62	0.07	6	1.45	1.78	**0.001**
	Grasslands	May	2.24	0.08	6	2.04	2.45	
	Heathlands	Jun	1.39	0.06	6	1.24	1.53	0.645
	Grasslands	Jun	1.35	0.06	6	1.20	1.49	
	Heathlands	Aug	1.00	0.06	6	0.84	1.15	0.483
	Grasslands	Aug	1.07	0.06	6	0.91	1.22	
	Heathlands	Oct	1.01	0.06	6	0.85	1.16	0.454
	Grasslands	Oct	1.08	0.07	6	0.92	1.24	
	Heathlands	Apr	1.40	0.07	6	1.22	1.58	**0.002**
	Grasslands	Apr	2.17	0.13	6	1.85	2.48	
Plant P (%)	Heathlands	May	0.15	0.02	6	0.09	0.21	**0.004**
	Grasslands	May	0.31	0.02	6	0.25	0.37	
	Heathlands	Jun	0.13	0.02	6	0.07	0.19	0.065
	Grasslands	Jun	0.21	0.02	6	0.15	0.26	
	Heathlands	Aug	0.10	0.02	6	0.05	0.16	**0.050**
	Grasslands	Aug	0.19	0.03	6	0.12	0.26	
	Heathlands	Oct	0.10	0.02	6	0.04	0.16	0.067
	Grasslands	Oct	0.17	0.02	6	0.11	0.23	

Response	Habitat type	Sampling date/period	Estimate	SE	*df*	Lower CL	Upper CL	*P*-value
	Heathlands	Apr	0.10	0.02	6	0.04	0.16	**0.036**
	Grasslands	Apr	0.20	0.03	6	0.14	0.27	
Observed faecal N (%)	Heathlands	May	1.60	0.05	6	1.49	1.71	**≤0.001**
	Grasslands	May	3.49	0.03	6	3.40	3.57	
	Heathlands	Jun	1.79	0.05	6	1.67	1.90	**≤0.001**
	Grasslands	Jun	2.43	0.03	6	2.35	2.51	
	Heathlands	Aug	2.04	0.21	6	1.51	2.56	0.210
	Grasslands	Aug	2.39	0.14	6	2.06	2.73	
	Heathlands	Oct	2.05	0.12	6	1.75	2.34	0.196
	Grasslands	Oct	2.27	0.09	6	2.04	2.49	
	Heathlands	Apr	2.05	0.41	6	1.05	3.05	0.056
	Grasslands	Apr	3.25	0.30	6	2.51	3.99	
Faecal P (%)	Heathlands	-	0.15	0.01	6	0.13	0.16	**≤0.001**
	Grasslands	-	0.57	0.05	6	0.44	0.71	
Dung dry matter (kg ha^{-1})	Heathlands	Apr–May	1.87	0.20	6	1.38	2.35	0.084
	Grasslands	Apr–May	1.34	0.16	6	0.95	1.74	
	Heathlands	May–Jun	1.00	0.15	6	0.63	1.36	0.070
	Grasslands	May–Jun	1.51	0.18	6	1.08	1.94	
	Heathlands	Jun–Aug	0.53	0.13	6	0.22	0.85	**≤0.001**
	Grasslands	Jun–Aug	1.86	0.15	6	1.50	2.23	
	Heathlands	Aug–Oct	1.10	0.17	6	0.69	1.51	0.029

Response	Habitat type	Sampling date/period	Estimate	SE	*df*	Lower CL	Upper CL	*P*-value
	Grasslands	Aug–Oct	1.90	0.22	6	1.35	2.44	
	Heathlands	Oct–Apr	7.73	0.55	6	6.38	9.08	**≤0.001**
	Grasslands	Oct–Apr	2.73	0.16	6	2.34	3.12	
N import (kg ha^{-1})	Heathlands	Apr–May	0.32	0.04	6	0.23	0.41	0.334
	Grasslands	Apr–May	0.38	0.04	6	0.28	0.48	
	Heathlands	May–Jun	0.18	0.03	6	0.11	0.25	**0.001**
	Grasslands	May–Jun	0.45	0.04	6	0.36	0.54	
	Heathlands	Jun–Aug	0.10	0.03	6	0.04	0.17	**≤0.001**
	Grasslands	Jun–Aug	0.45	0.03	6	0.37	0.53	
	Heathlands	Aug–Oct	0.25	0.03	6	0.17	0.33	**0.014**
	Grasslands	Aug–Oct	0.44	0.05	6	0.33	0.56	
	Heathlands	Oct–Apr	1.52	0.12	6	1.23	1.81	**0.001**
	Grasslands	Oct–Apr	0.73	0.04	6	0.63	0.83	
P import (kg ha^{-1})	Heathlands	Apr–May	0.03	0.00	6	0.03	0.04	**≤0.001**
	Grasslands	Apr–May	0.11	0.01	6	0.10	0.13	
	Heathlands	May–Jun	0.02	0.00	6	0.01	0.02	**≤0.001**
	Grasslands	May–Jun	0.10	0.01	6	0.08	0.11	
	Heathlands	Jun–Aug	0.01	0.00	6	0.00	0.02	**≤0.001**
	Grasslands	Jun–Aug	0.09	0.01	6	0.08	0.10	
	Heathlands	Aug–Oct	0.04	0.00	6	0.03	0.05	**≤0.001**
	Grasslands	Aug–Oct	0.12	0.01	6	0.11	0.13	

Response	Habitat type	Sampling date/period	Estimate	SE	*df*	Lower CL	Upper CL	*P*-value
	Heathlands	Oct–Apr	0.14	0.01	6	0.12	0.17	**≤0.001**
	Grasslands	Oct–Apr	0.22	0.01	6	0.20	0.25	
Forage removal (kg ha^{-1} d^{-1})	Heathlands	Apr–May	68.01	35.36	6	-18.52	154.53	**0.012**
	Grasslands	Apr–May	321.36	62.22	6	169.12	473.60	
	Heathlands	May–Jun	0.99	7.50	6	-17.36	19.35	0.442
	Grasslands	May–Jun	12.34	11.58	6	-15.99	40.67	
	Heathlands	Jun–Aug	0.04	1.27	6	-3.06	3.14	**≤0.001**
	Grasslands	Jun–Aug	99.92	9.40	6	76.92	122.92	
	Heathlands	Aug–Oct	11.68	25.42	6	-50.52	73.87	0.622
	Grasslands	Aug–Oct	27.71	17.45	6	-14.99	70.40	
	Heathlands	Oct–Apr	434.17	124.21	6	130.24	738.09	0.164
	Grasslands	Oct–Apr	866.00	242.25	6	273.23	1458.78	
N export (kg ha^{-1})	Heathlands	Apr–May	1.73	0.93	6	-0.55	4.01	**0.014**
	Grasslands	Apr–May	8.63	1.78	6	4.28	12.99	
	Heathlands	May–Jun	0.29	0.23	6	-0.28	0.85	0.425
	Grasslands	May–Jun	0.04	0.18	6	-0.40	0.47	
	Heathlands	Jun–Aug	0.01	0.06	6	-0.13	0.15	**0.001**
	Grasslands	Jun–Aug	1.30	0.21	6	0.78	1.82	
	Heathlands	Aug–Oct	0.13	0.24	6	-0.46	0.71	0.625
	Grasslands	Aug–Oct	0.28	0.19	6	-0.18	0.75	
	Heathlands	Oct–Apr	8.31	2.93	6	1.14	15.47	0.153

Response	Habitat type	Sampling date/period	Estimate	SE	*df*	Lower CL	Upper CL	*P*-value
	Grasslands	Oct–Apr	2.98	1.41	6	-0.47	6.44	
P export (kg ha^{-1})	Heathlands	Apr–May	0.51	0.17	6	0.10	0.92	**0.010**
	Grasslands	Apr–May	0.84	0.18	6	0.39	1.29	
	Heathlands	May–Jun	0.10	0.09	6	-0.13	0.33	**0.010**
	Grasslands	May–Jun	0.43	0.12	6	0.13	0.72	
	Heathlands	Jun–Aug	0.13	0.04	6	0.02	0.23	**0.010**
	Grasslands	Jun–Aug	0.45	0.09	6	0.24	0.67	
	Heathlands	Aug–Oct	0.06	0.06	6	-0.09	0.21	**0.010**
	Grasslands	Aug–Oct	0.39	0.10	6	0.15	0.62	
	Heathlands	Oct–Apr	0.85	0.13	6	0.53	1.17	**0.010**
	Grasslands	Oct–Apr	1.18	0.15	6	0.81	1.55	

Table S3 Aboveground net primary productivity (ANPP, kg ha^{-1}; Riesch et al. 2019) in heathlands and grasslands and the associated amount of nitrogen (N) and phosphorus (P) in the vegetation biomass based on plant N and P concentrations (cf. Table S1), as well as the net nutrient removal by wild red deer (cf. Table 2, main text) expressed as absolute mean value and as percent of the nutrient quantity in the ANPP for the study year April 2015–April 2016

		ANPP (kg ha^{-1})			N in ANPP (kg ha^{-1})			Net N removal		P in ANPP (kg ha^{-1})			Net P removal	
Habitat type	n	Mean	SD	SE	Mean	SD	SE	Mean (kg ha^{-1})	Percent (%)	Mean	SD	SE	Mean (kg ha^{-1})	Percent (%)
Heathlands	6	2227.97	1251.38	510.87	28.82	15.84	6.47	13.89	48.20	2.59	1.45	0.59	1.01	39.04
Grasslands	8	4177.81	1167.58	412.80	73.95	17.95	6.35	29.58	40.00	9.70	2.65	0.94	3.30	34.06

Table S4 Approximation of annual export of nitrogen (N) and phosphorus (P) through culled red deer based on the number (hunting bag) and weight of animals shot in the hunting districs comprising our sampling sites during the study year (starting from April 2015) documented by the Federal Forestry Administration. As carcasses were weighted without head and entrails, we have multiplied the slaughter weight by 1.25. For the few cases in which carcass weight data were not available, we have added the mean over the available data to estimate the total weight of all culled animals

Hunting district				Culled red deer weight (kg)				Nitrogen export with culled red deer[c]			Phosphorus export with culled red deer[d]		
District acronym	District area (ha)	Sampling sites habitat type	N sampling sites	Red deer hunting bag	Mean[a]	Sum[a]	Estimated total[b]	N (kg)	N (kg ha^{-1})	N (g ha^{-1})	P (kg)	P (kg ha^{-1})	P (g ha^{-1})
NB	2646.90	Grasslands	2	200	61.31	11342.63	12262.30	343.34	0.13	129.72	122.62	0.05	46.33
LH	2512.84	Grasslands	2	198	61.73	12099.38	12222.84	342.24	0.14	136.20	122.23	0.05	48.64
ST	3858.27	Heathlands	3	289	53.88	15032.88	15625.57	437.52	0.11	113.40	156.26	0.04	40.50
SB	2707.26	Heathlands	1	219	58.31	11837.00	12769.97	357.56	0.13	132.07	127.70	0.05	47.17

[a] Weight data were missing for some animals, so that mean and sum were calculated based on n=185 in NB, n=197 in LH, n=278 in ST and n=219 in SB

[b] To approximate the total weight of all culled animals, we added the mean weight for each animal for which weight data were not available

[c] Based on 2.8% N in body tissue (Whitehead 2000)

[d] Based on 1.0% P in body tissue (Whitehead 2000)

Table S5 Model terms and associated numerator ($df_{(num)}$) and denominator degrees of freedom ($df_{(den)}$), *F*- and *P*-values of marginal Wald test in the linear mixed effects model analysing differences between observed and expected faecal N concentration. The conditional ($R_{(c)}^2$) and marginal ($R_{(m)}^2$) coefficients of determination express the variance explained by fixed and random effects combined and the variance explained only by fixed effects (Nakagawa et al. 2017)

Response	Model term	$df_{(num)}$	$df_{(den)}$	*F*-value	*P*-value	$R_{(m)}^2$	$R_{(c)}^2$
Faecal N	Data type[a]	1	51	126.79	≤0.001	0.98	0.99
	Habitat type[b]	1	6	52.01	≤0.001		
	Sampling date[c]	4	51	84.66	≤0.001		
	Data type × Habitat type	1	51	34.00	≤0.001		
	Data type × Sampling date	4	51	4.18	0.005		
	Habitat type × Sampling date	4	51	107.35	≤0.001		
	Data type × Habitat type × Sampling date	4	51	43.31	≤0.001		

[a] Two-level factor (observed faecal N concentration, expected faecal N based on hand-pluck sample plant N concentration)

[b] Two-level factor (heathlands, grasslands)

[c] Five-level factor (May, Jun, Aug, Oct, Apr)

Table S6 Estimated marginal means and related standard errors (SE), degrees of freedom (*df*) and 95%-confidence limits (CL) from the linear mixed effects model for faecal N concentration (the N concentration observed in red deer dung or expected based on hand-pluck sample plant N concentration). *P*-values are associated with the contrasts between observed and expected faecal N concentration within habitat and sampling date. *P*-values <0.05 are printed in bold

Response	Habitat type	Sampling date	Data type	Estimate	SE	*df*	Lower CL	Upper CL	*P*-value
Faecal N (%)	Heathlands	May 2015	Observed	1.58	0.04	6	1.48	1.68	**≤0.001**
	Heathlands	May 2015	Expected	1.73	0.04	6	1.64	1.82	
	Grasslands	May 2015	Observed	3.47	0.04	6	3.37	3.56	**≤0.001**
	Grasslands	May 2015	Expected	2.02	0.04	6	1.93	2.11	
	Heathlands	June 2015	Observed	1.79	0.04	6	1.68	1.89	**≤0.001**
	Heathlands	June 2015	Expected	1.61	0.03	6	1.53	1.69	
	Grasslands	June 2015	Observed	2.43	0.04	6	2.32	2.53	**≤0.001**
	Grasslands	June 2015	Expected	1.59	0.03	6	1.51	1.67	
	Heathlands	August 2015	Observed	2.03	0.23	6	1.47	2.58	**0.010**
	Heathlands	August 2015	Expected	1.42	0.03	6	1.36	1.49	
	Grasslands	August 2015	Observed	2.39	0.20	6	1.91	2.88	**≤0.001**
	Grasslands	August 2015	Expected	1.46	0.03	6	1.40	1.53	
	Heathlands	October 2015	Observed	2.05	0.12	6	1.75	2.34	**≤0.001**
	Heathlands	October 2015	Expected	1.43	0.03	6	1.36	1.50	
	Grasslands	October 2015	Observed	2.27	0.12	6	1.97	2.56	**≤0.001**
	Grasslands	October 2015	Expected	1.46	0.03	6	1.39	1.53	
	Heathlands	April 2016	Observed	2.05	0.35	6	1.19	2.91	0.240

Response	Habitat type	Sampling date	Data type	Estimate	SE	*df*	Lower CL	Upper CL	*P*-value
	Heathlands	April 2016	Expected	1.64	0.04	6	1.54	1.73	
	Grasslands	April 2016	Observed	3.25	0.35	6	2.39	4.11	**0.001**
	Grasslands	April 2016	Expected	2.06	0.04	6	1.97	2.15	

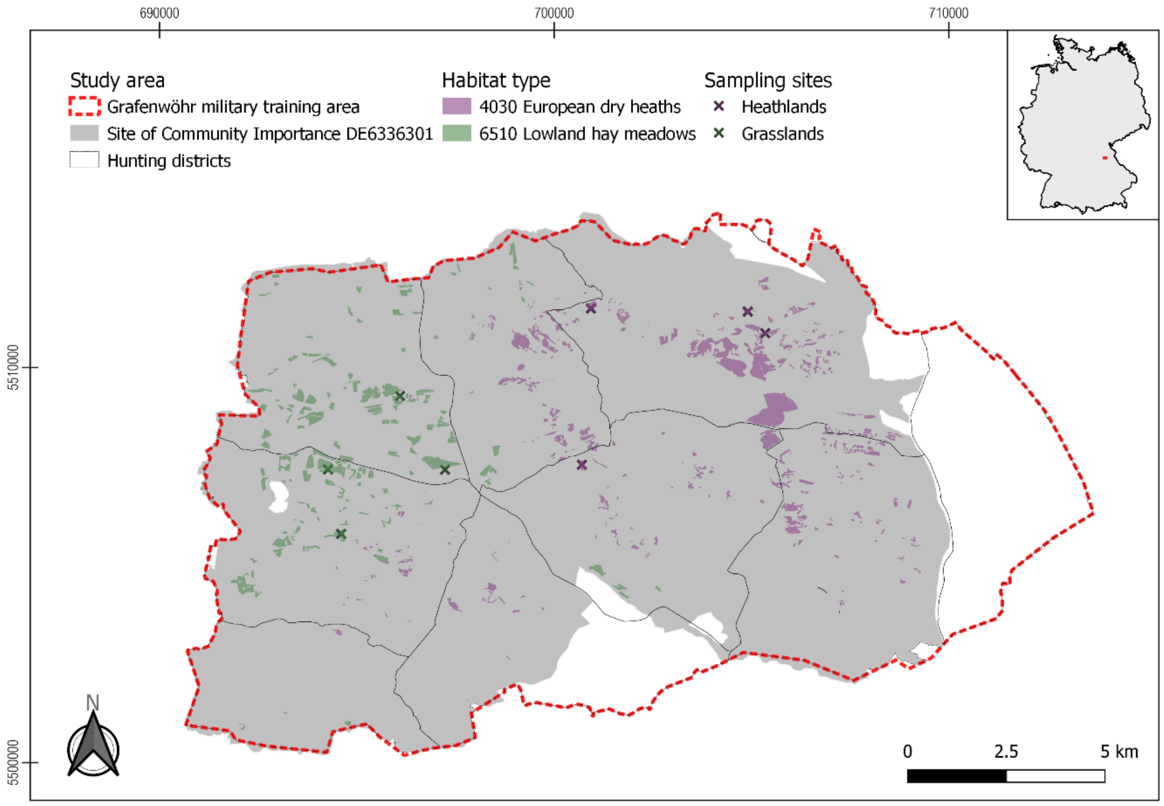

Fig. S1 Map of the study area Grafenwöhr military training area with the extent of the focal habitat types protected under the European Habitats Directive (BImA – Sparte Bundesforst 2019) within the Site of community Importance DE6336301 (Bayerisches Landesamt für Umwelt) and the locations of the sampling sites. The small map in the upper right corner shows the location of the study area in Germany.

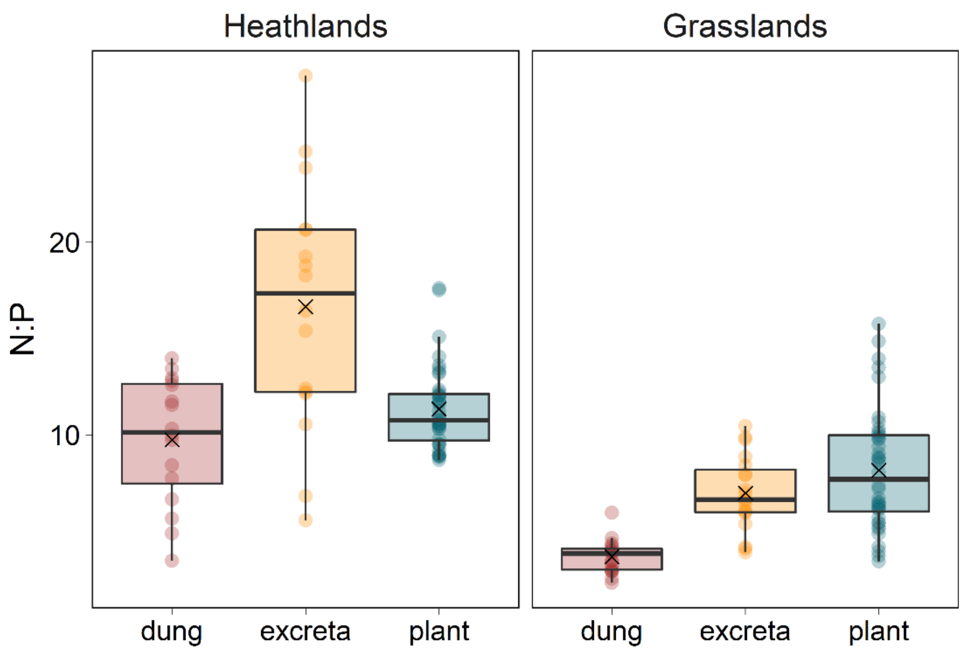

Fig. S2 Nitrogen to phosphorus ratio (N:P) of dung and excreta (including N in both dung and urine) of wild red deer and of vegetation in heathlands and grasslands in Grafenwöhr military trainings area, Germany. The cross symbol indicates the arithmetic mean; circles represent observations

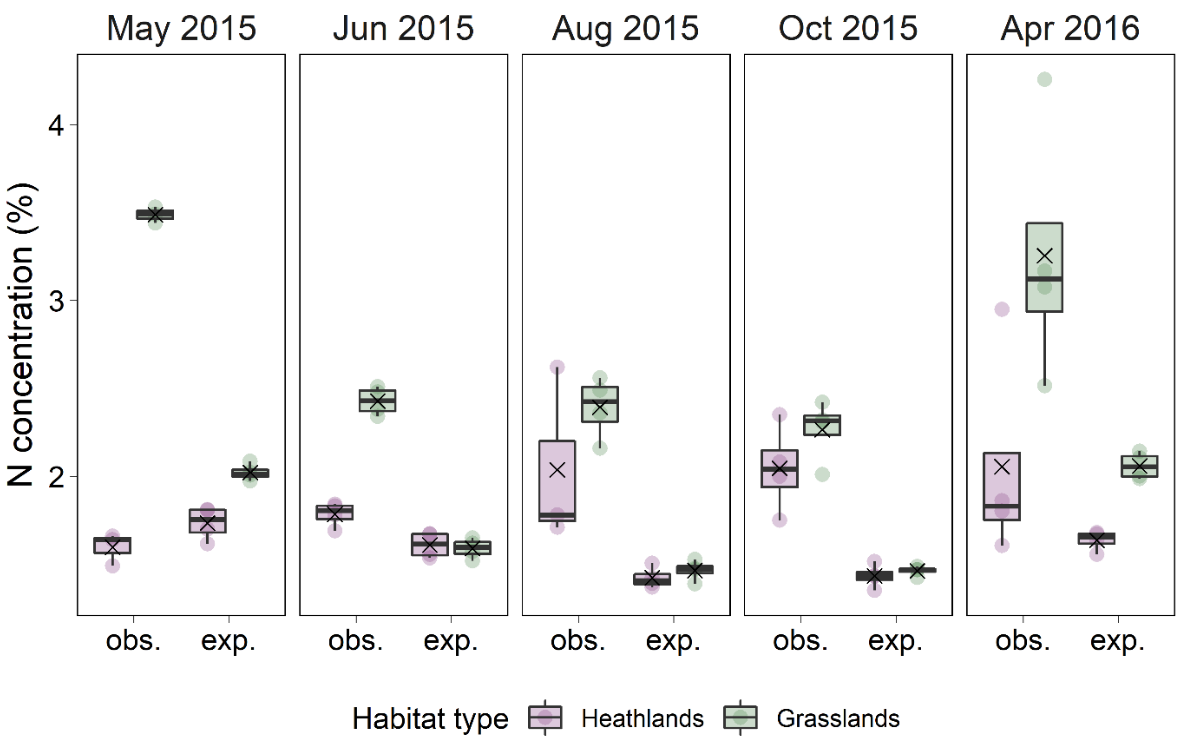

Fig. S3 Observed faecal N concentration (obs.) in dung of wild red deer collected in heathlands and grasslands in Grafenwöhr military training area, Germany, and expected faecal N concentration (exp.) predicted based on plant N concentration in hand-pluck samples. The cross symbol indicates the arithmetic mean; circles represent observations

References

Arturo G-C, Diana G, Ramón L-OJ, Gregorio M, Jordi B, Ignasi M, et al (2015) Gastrointestinal nematodes and dietary fibre: Two factors to consider when using FN for wildlife nutrition monitoring. Ecol Indic 52:161–169. https://doi.org/10.1016/j.ecolind.2014.11.020

Christianson D, Creel S (2010) A nutritionally mediated risk effect of wolves on elk. Ecology 91:1184–1191. https://doi.org/10.1890/09-0221.1

Čupić S, García AJ, Holá M, Ceacero F (2021) Evaluation of factors inducing variability of faecal nutrients in captive red deer under variable demands. Sci Rep 11:2394. https://doi.org/10.1038/s41598-021-81908-y

Gordon IJ (2003) Browsing and grazing ruminants: are they different beasts? For Ecol Manag 181:13–21

Hofland-Zijlstra JD, Berendse F (2009) The effect of nutrient supply and light intensity on tannins and mycorrhizal colonisation in Dutch heathland ecosystems. Plant Ecol 201:661–675. https://doi.org/10.1007/s11258-008-9554-3

Hofmann RR (1989) Evolutionary steps of ecophysiological adaptation and diversification of ruminants: a comparative view of their digestive system. Oecologia 78:443–457

Langvatn R, Hanley TA (1993) Feeding-patch choice by red deer in relation to foraging efficiency. Oecologia 95:164–170

Leslie DM, Bowyer RT, Jenks JA (2008) Facts from Feces: Nitrogen Still Measures Up as a Nutritional Index for Mammalian Herbivores. J Wildl Manag 72:1420–1433. https://doi.org/10.2193/2007-404

Moen R, DelGiudice GD (1997) Simulating Nitrogen Metabolism and Urinary Urea Nitrogen: Creatinine Ratios in Ruminants. J Wildl Manag 61:881–894. https://doi.org/10.2307/3802197

Nakagawa S, Johnson PCD, Schielzeth H (2017) The coefficient of determination R2 and intra-class correlation coefficient from generalized linear mixed-effects models revisited and expanded. J R Soc Interface 14:20170213. https://doi.org/10.1098/rsif.2017.0213

Riesch F, Stroh HG, Tonn B, Isselstein J (2018) Soil pH and phosphorus drive species composition and richness in semi-natural heathlands and grasslands unaffected by twentieth-century agricultural intensification. Plant Ecol Divers 11:239–253

Riesch F, Tonn B, Meißner M, Balkenhol N, Isselstein J (2019) Grazing by wild red deer: Management options for the conservation of semi-natural open habitats. J Appl Ecol 56:1311–1321. https://doi.org/10.1111/1365-2664.13396

Semiadi G, Barry T, Muir P, Hodgson J (1995) Dietary Preferences of Sambar (Cervus unicolor) and Red Deer (Cervus elaphus) Offered Browse, Forage Legume and Grass Species. J Agric Sci 125:99–107. https://doi.org/10.1017/S0021859600074554

Verheyden-Tixier H, Renaud P-C, Morellet N, Jamot J, Besle J-M, Dumont B (2008) Selection for nutrients by red deer hinds feeding on a mixed forest edge. Oecologia 156:715–726

Whitehead DC (2000) Nutrient elements in grassland: soil-plant-animal relationships. CABI Pub, Wallingford, Oxon, UK; New York, NY
